# A Comparative Study of Topical Treatments for the Management of Chronic Leg Ulcers in a Multi-Center Cohort

**DOI:** 10.7759/cureus.104830

**Published:** 2026-03-07

**Authors:** Ayman Fakhry, Sohiel Nagib, Abdullah H Al-Mallah, Eslam M Barsim, Ahmed Khalf, Ahmed Abouelseoud, Mahmoud Moner, Omar Gad, Omar Alnadi, Muhammad Jabr, Ziad M Ghazy, Yousuf Abuelkhair, Marwan E Makayed, Mohamed Farrag, Sergiu-Ciprian Matei, Adriana Mocain, Alina Mirsu, Mohamed Soliman, Ayman Zyada

**Affiliations:** 1 Vascular Surgery, Egyptian Military Medical Academy, Alexandria, EGY; 2 Vascular Surgery, Royal Vascular Center, Alexandria, EGY; 3 Medical Education, Maastricht University, Netherlands, NLD; 4 Medical Education, Al-Azhar University, Cairo, EGY; 5 Vascular Surgery, Al-Azhar University, Cairo, EGY; 6 Vascular Surgery, Menofia University Faculty of Medicine, Menofia, EGY; 7 Vascular Surgery, Aswan University, Aswan, EGY; 8 Vascular Surgery, Alexandria Main University Hospital, Alexandria, EGY; 9 Surgery and Medicine, Al-Ahrar Teaching Hospital, Al-Sharqia, EGY; 10 Vascular Surgery, Abou Qir General Hospital, Alexandria, EGY; 11 General Surgery, Abo Qir General Hospital, Alexandria, EGY; 12 Vascular Surgery, Abu Qir General Hospital, Alexandria, EGY; 13 Surgery, Alexandria Main University Hospital, Alexandria, EGY; 14 Faculty of Medicine, Alexandria University, Alexandria, EGY; 15 Abdominal Surgery and Phlebology Research Center, "Victor Babeș” University of Medicine and Pharmacy, Timisoara, ROU; 16 Vascular Surgery, Transilvania Hospital Cluj-Napoca, Cluj-Napoca, ROU; 17 Vascular Surgery, Deva County Emergency Hospital, Deva, ROU; 18 Vascular Surgery, Suez Canal University, Suez, EGY; 19 Vascular Surgery, Aberdeen Royal Infirmary, Aberdeen, GBR

**Keywords:** chronic leg ulcers, enzymatic wound care, hyaluronic acid, topical wound therapy, venous leg ulcers, wound care management, wound healing

## Abstract

Background

Chronic leg ulcers represent a significant clinical and economic burden due to prolonged healing times, frequent outpatient visits, and high recurrence rates. Topical wound care is widely used; however, comparative evidence supporting specific topical agents remains limited, and inappropriate or prolonged use may delay healing or increase complications. This multi-center study aimed to compare the effectiveness of three commonly used topical treatment strategies in the management of chronic leg ulcers.

Methods

A total of 125 patients with chronic leg ulcers of more than four weeks’ duration were enrolled across multiple centers between July 1, 2025, and December 30, 2025. Two patients withdrew early and were excluded, leaving 123 patients for final analysis. Patients were allocated into three treatment groups: 22 patients received antimicrobial topical therapy, 33 patients received hyaluronic acid-based therapy, and 68 patients received a combined enzymatic topical formulation. The primary endpoint was complete ulcer healing within four months, defined as full epithelialization without discharge.

Results

Complete healing occurred in 45.45% of patients in the antimicrobial group, 75.76% in the hyaluronic acid group, and 94% in the combined formulation group. Mean healing time was 9.73 ± 0.09 weeks, 9.11 ± 1.02 weeks, and 5.8 ± 0.12 weeks, respectively. Logistic regression demonstrated significantly higher odds of complete healing in the hyaluronic acid group (OR 3.75, p = 0.022) and the combined formulation group (OR 4.63, p = 0.002) compared with antimicrobial therapy alone.

Conclusion

The choice of topical dressing significantly influences healing outcomes in patients with chronic leg ulcers. A combined enzymatic, antimicrobial, and moisture-retentive formulation was associated with faster healing and higher rates of complete ulcer closure compared with antimicrobial or hyaluronic acid therapy alone. These findings support a more individualized, evidence-based approach to topical wound care as part of comprehensive chronic leg ulcer management.

## Introduction

Chronic leg ulcers (CLUs) constitute a significant global health burden, accounting for substantial morbidity, impaired quality of life, and escalating healthcare expenditures. Their management is often complex, necessitating prolonged treatment courses, frequent outpatient encounters, and considerable utilization of healthcare resources. Among current therapeutic strategies, topical wound care remains widely employed due to its accessibility and its perceived role in optimizing the local wound environment. Epidemiological data, though heterogeneous, indicate that CLUs, particularly venous leg ulcers (VLUs), are not rare in many populations. A recent systematic review and meta-analysis estimated a pooled prevalence of VLUs of approximately 0.32% and a pooled incidence of approximately 0.17% per year [1]. Other population-based studies have reported chronic lower-limb ulcer prevalence ranging from 0.12% to 1.1% of the general adult population, depending on definitions and methods [2]. Prevalence rises markedly with age, reaching 3-5% in individuals older than 65 years in some cohorts [3].

However, the clinical value of many topical agents remains insufficiently characterized. Existing products vary widely in composition, cost, and mechanism of action, yet robust comparative evidence is limited. Furthermore, the use of topical therapies is not without drawbacks. Many formulations are associated with under-recognized but clinically relevant adverse effects, including pain, skin maceration, contact dermatitis, and allergic reactions. In addition, an overemphasis on topical interventions may inadvertently divert attention from systemic or etiological treatments that are often more effective and more cost-efficient in promoting ulcer resolution.

This gap in evidence-based guidance is particularly evident in the management of CLUs within the Egyptian population, where practice patterns vary considerably and standardized protocols remain limited. Recognizing this unmet need, the Egyptian Venous Forum Research Team has identified the evaluation of topical wound therapies as a priority area requiring rigorous scientific investigation. The present multicenter study was therefore designed to comparatively assess the efficacy and safety of different topical treatment modalities in patients with CLUs. By generating high-quality clinical data, this study aims to inform future clinical practice, support the development of rational application policies, and contribute to reducing therapeutic ambiguity in the management of CLUs.

Recognizing this unmet need, the Egyptian Venous Forum Research Team designed this multicenter, quasi-experimental comparative cohort study to evaluate commonly used topical treatment strategies in CLUs. The primary objective of the study was to compare complete ulcer healing rates and time to healing within 16 weeks among three topical treatment modalities. Secondary objectives included evaluating factors influencing healing outcomes, including baseline ulcer size, comorbidities, ulcer etiology, and the relationship between initial ulcer dimensions and healing duration.

We hypothesized that a combined enzymatic, antimicrobial, and moisture-retentive topical formulation would demonstrate superior healing outcomes compared with antimicrobial or hyaluronic acid therapy alone. By generating comparative clinical data, this study aims to inform evidence-based topical therapy selection within the broader framework of comprehensive CLU management.

## Materials and methods

A total of 125 patients with CLUs of more than four weeks’ duration were enrolled from multiple study centers between July 1, 2025, and December 30, 2025. This multi-center study was conducted in nine vascular surgery units across Egypt and Romania. Participating Egyptian centers included the Department of Vascular Surgery, Royal Vascular Center, Alexandria; the Vascular Surgery Department, Menofia University Faculty of Medicine, Menofia; the Department of Vascular Surgery, Aswan University, Aswan; the Vascular Surgery Unit, Abou Qir General Hospital, Alexandria; the Department of Vascular Surgery, Mansora University, Mansora; and the Vascular Surgery Department, Suez Canal University, Suez. Participating Romanian centers included the Abdominal Surgery and Phlebology Research Center, “Victor Babeș” University of Medicine and Pharmacy, Timișoara; the Vascular Surgery Department, Transilvania Hospital Cluj‑Napoca, Cluj‑Napoca; and the Vascular Surgery Department, Deva County Emergency Hospital, Deva.

Patients were assigned to one of three treatment groups. Group A received wound irrigation with normal saline (0.9% sodium chloride), followed by chlorhexidine 4% antiseptic solution (Hibiwash, Mölnlycke Health Care AB, Gothenburg, Sweden), application of an antimicrobial cream (Fucidin cream, fusidic acid 2%, LEO Pharma A/S, Ballerup, Denmark), and sterile dressing. Group B received wound irrigation with normal saline, chlorhexidine 4% antiseptic solution, application of a hyaluronic acid-based cream (Hyalugel, hyaluronic acid, Ricerfarma S.r.l., Milan, Italy), and sterile dressing. Group C received wound irrigation with normal saline, chlorhexidine 4% antiseptic solution, and a combined enzymatic topical formulation containing antimicrobial agents, alginate for debridement, hyaluronic acid, and moisture-retentive components (Zymalge gel, Pharma Mart Group, Giza, Egypt), followed by sterile dressing.

This study was designed as a quasi-experimental, time-sequential multicenter cohort study. Treatment allocation was performed using a predefined time-based sequential protocol, whereby each participating center applied one topical treatment strategy for a fixed three-month period before transitioning to the next protocol.

**Figure 1:**
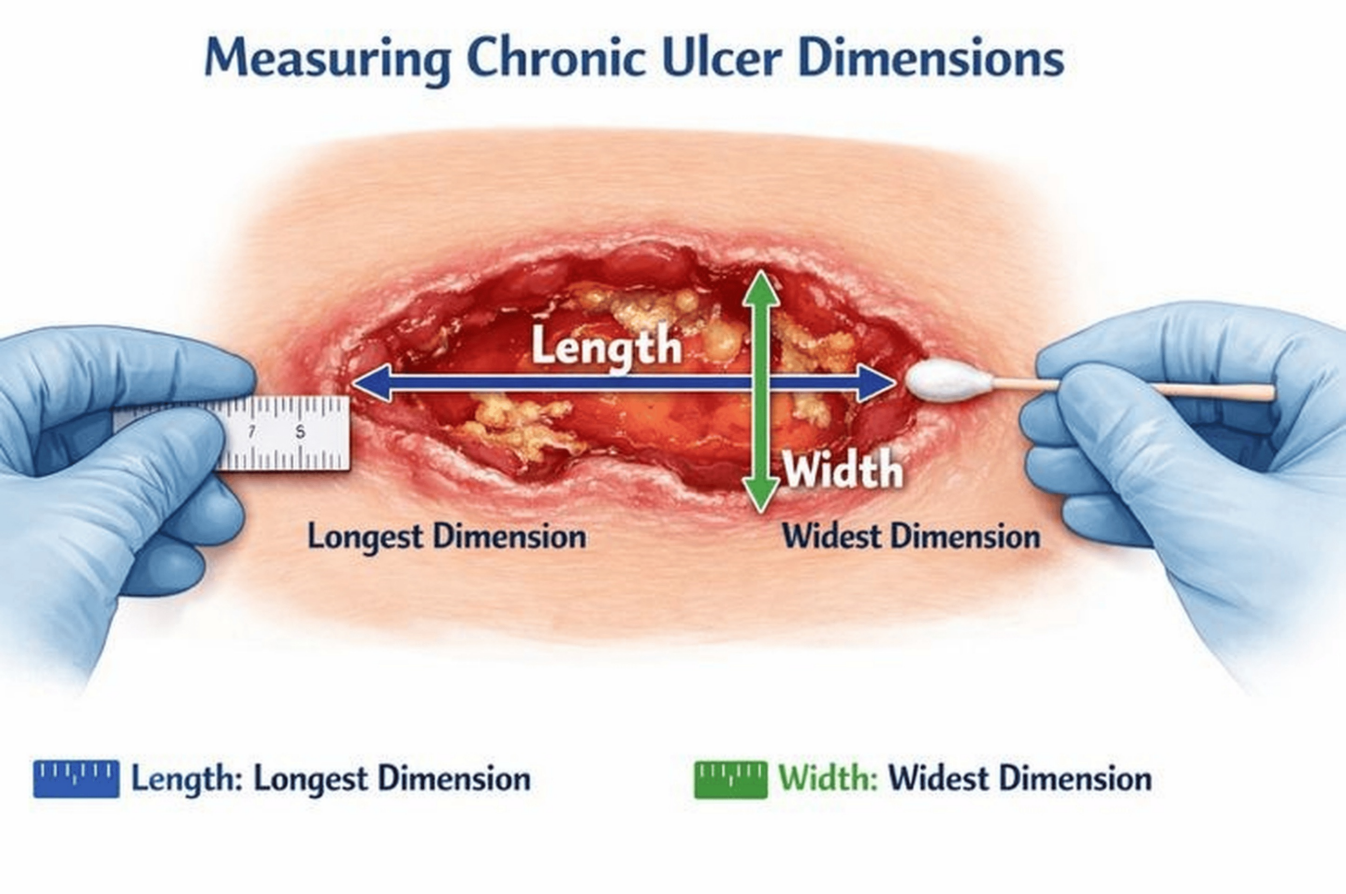
Measuring chronic ulcer dimensions Schematic illustration demonstrating standardized measurement of chronic ulcer dimensions using longitudinal and transverse diameters. The figure is an original illustration created by the authors specifically for this study and is not reproduced or adapted from any copyrighted source. The figure was created using photography, with subsequent editing performed using Adobe Photoshop and Canva for layout enhancement and visual adjustments.

Written informed consent was obtained from all participants prior to enrolment, including consent for clinical photography and anonymized publication of images. Following consent, all patients underwent comprehensive clinical assessment, including detailed history-taking, general physical examination, and ulcer evaluation with measurement (Figure 1) and digital documentation. Ulcers were classified according to the Texas Wound Classification system [4], as shown in Table 1.

**Table 1:** Texas Wound Classification This table represents the University of Texas classification of foot ulcers [4]. Ulcers are categorized by grade (0–3) based on depth of tissue involvement and by stage (A–D) based on the presence of infection and/or ischemia. Stages include A (no infection or ischemia), B (infection), C (ischemia), and D (both infection and ischemia). The table was created by the authors using information from the cited source

Stage	Grade 0	Grade 1	Grade 2	Grade 3
A	Pre-ulcerative lesions; no skin break	Superficial wound; no penetration	Wound penetrating tendon or capsule	Wound penetrating bone or joint
B	With infection	With infection	With infection	With infection
C	With ischemia	With ischemia	With ischemia	With ischemia
D	With infection and ischemia	With infection and ischemia	With infection and ischemia	With infection and ischemia

Vascular assessment was systematically performed in all patients, and etiological correction was undertaken when indicated prior to or in parallel with topical treatment. Additional assessments included ankle-brachial index measurement, venous system evaluation, bony assessment for deformity detection, routine laboratory investigations, and radiological evaluation when clinically indicated.

Eligible patients were adults aged 18 to 70 years with controlled diabetes mellitus (HbA1c ≤ 8.0% within three months prior to enrolment) and hypertension. Exclusion criteria included heart failure, uremia, malignancy, and collagen vascular diseases. The primary endpoint was complete ulcer healing within four months, defined as full epithelialization without discharge. The secondary endpoint was incomplete ulcer healing within the recommended four-month study period or patient withdrawal from the study. Dressings were applied once daily in all groups unless excessive exudate required additional change. Wound cleansing and dressing application followed a standardized protocol across centers to ensure consistency. Patients were evaluated weekly during the follow-up period, with wound measurements and documentation performed at each visit.

A formal a priori sample size calculation was not performed for this study. The investigation was designed as a pragmatic, multicenter comparative cohort reflecting real-world clinical practice. Patient recruitment was therefore based on the number of eligible patients presenting to the participating centers within the predefined study period. This approach was chosen to ensure feasibility, minimize selection bias, and enhance external validity.

Despite the absence of a predefined sample size calculation, the final cohort of patients provided sufficient data to allow meaningful comparative statistical analyses between treatment groups, including one-way analysis of variance (ANOVA) and logistic regression modeling for the primary outcome of complete ulcer healing.

Statistical analysis was performed using Stata software, version 12.0 (StataCorp LLC, College Station, TX, USA). Continuous variables were expressed as mean ± standard deviation and compared using ANOVA. Categorical variables were compared using the two-proportion Z-test.

To evaluate the association between treatment group and complete ulcer healing, binary logistic regression analysis was performed using the antimicrobial topical therapy group as the reference category. Results were reported as regression coefficients with corresponding standard errors, z-statistics, p-values, and 95% confidence intervals.

Correlation between initial ulcer size and healing duration was assessed using the Pearson correlation coefficient. A p-value of <0.05 was considered statistically significant.

## Results

This study analyzed outcomes in patients with CLUs, focusing on demographic characteristics, comorbidities, ulcer features, etiology, treatment arm comparison, and healing outcomes. The study cohort consisted of 125 patients. Two patients (0.8%) withdrew within the first few days and did not complete the study period. The remaining patients were allocated into three treatment groups. The first group (antimicrobial topical therapy) included 22 patients, the second group (hyaluronic acid-based therapy) included 33 patients, and the third group (mixed combination topical therapy) included 68 patients.

The unequal group sizes resulted from variability in patient recruitment during sequential treatment phases across centers. Recruitment was consecutive, and no enrolment cap was imposed during each three-month treatment block. Higher patient volume during the period in which the combined formulation was applied resulted in a larger group 3 sample. Allocation was time-based and not influenced by ulcer etiology or clinical severity. This reflects the pragmatic real-world design of the study rather than intentional allocation imbalance.

The mean age of the study population was 49.65 ± 0.9 years, with 70 patients being male (56.9%) and 53 being female (43.1%). The mean duration of ulcers prior to initiation of the dressing protocol was 20.28 ± 0.6 weeks. Mean ulcer duration before treatment was 22.2 ± 0.2 weeks in the first group, 20.2 ± 0.3 weeks in the second group, and 18.9 ± 0.7 weeks in the third group (Table 2).

**Table 2:** Average duration of the ulcer before initiation of dressing protocol

N	Group	Total patients	Duration before treatment (weeks, mean ± SD)
1	Antimicrobial topical therapy	22 patients	22.2 + 0.2
2	Hyaluronic acid–based therapy	33 patients	20.2 + 0.3
3	Mixed combination topical therapy	68 patients	18.9 + 0.7

**Figure 2:**
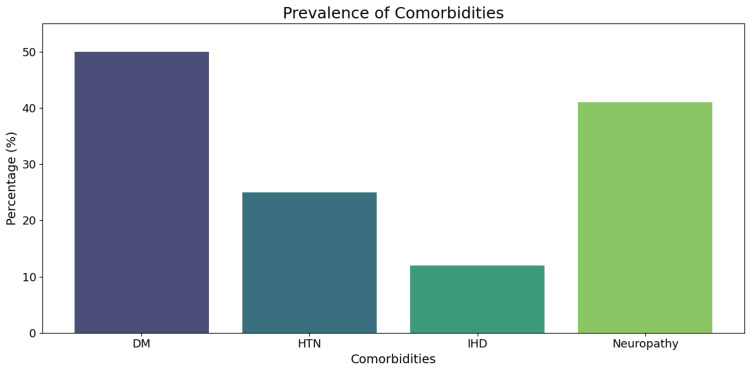
Prevalence of key comorbidities among the study population (n = 123) Prevalence of key comorbidities among the study population. The bar chart illustrates the percentage distribution of major comorbid conditions, including DM, HTN, IHD, and peripheral neuropathy, which were: DM: 61 patients (49.59%) HTN: 31 patients (25.20%) IHD: 15 patients (12.20%) Peripheral neuropathy: 51 patients (41.46%) DM, diabetes mellitus; HTN, hypertension; IHD, ischemic heart disease

A substantial proportion of patients presented with associated comorbidities, most notably diabetes mellitus and peripheral neuropathy (Figure 2).

The mean initial ulcer size across the study population was 22.11 ± 0.4 cm². Mean initial ulcer size was 18.9 ± 0.6 cm² in the first group, 23.06 ± 0.4 cm² in the second group, and 21.5 ± 0.7 cm² in the third group.

Ulcers were classified according to the Texas Wound Classification system, as shown in Table 3.

**Table 3:** Texas Wound Classification stages and grades The first group represents patients treated with antimicrobial topical therapy, the second group represents patients treated with hyaluronic acid–based topical therapy, and the third group represents patients treated with mixed combination topical therapy (enzymatic, antimicrobial, alginate, and hyaluronic acid formulation).

Classification	First group	Second group	Third group
Stage A	9 patients	13 patients	32 patients
Stage B	5 patients	8 patients	18 patients
Stage C	7 patients	10 patients	13 patients
Stage D	1 patient	2 patients	5 patients
Grade 1	7 patients	14 patients	16 patients
Grade 2	5 patients	9 patients	14 patients
Grade 3	10 patients	10 patients	38 patients

Clinical evidence of infection was present in 116 (95.12%) patients. Signs of systemic involvement were observed in 15 (12.5%) patients, who received systemic antibiotics according to culture and sensitivity testing.

The primary ulcer etiologies were venous, ischemic, pressure-related, traumatic, or mixed pathology. Venous ulcers accounted for 54.15% of cases, ischemic ulcers for 20.57%, pressure ulcers for 7%, traumatic ulcers for 3%, and mixed pathology ulcers for 15.28%. Targeted correction or management of the underlying etiology was achieved in 74.72% of cases.

**Figure 3:**
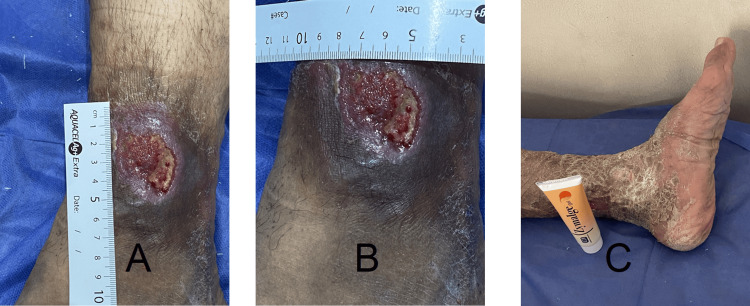
Healed venous ulcer The images show healing of the venous ulcer in a male patient (49 years, group 3). (A) Longitudinal diameter of venous ulcer at the start of treatment. (B) Transverse diameter of the venous ulcer at the start of treatment. (C) Completely healed venous ulcer.

Venous ulcers represented the most common etiology (54%). Identified venous causes included incompetent perforators, refluxing great saphenous vein, refluxing small saphenous vein, prior deep vein thrombosis with deep venous reflux, iliac vein compression, and mixed reflux patterns. Management strategies included compression therapy, great saphenous vein ablation in nineteen patients (15%), and sclerotherapy in six patients (5%). Representative healing of venous ulcers is shown in Figure 3.

**Figure 4:**
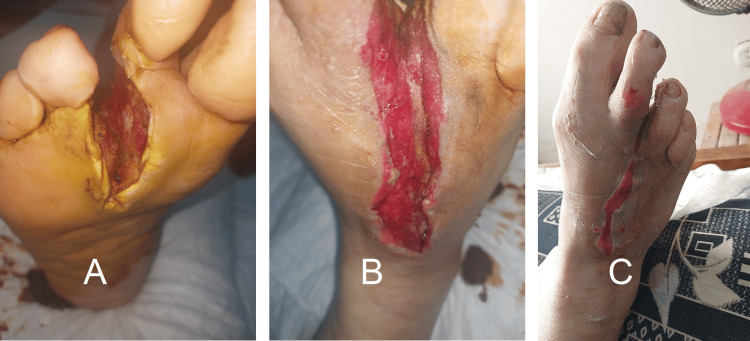
Arterial ulcer healing after tibial angioplasty and minor amputation The photo shows stages of arterial ulcer healing in a male patient (44 years, group 2). (A) Chronic foot wound after revascularization and minor amputation. (B) Wound healing after four weeks of treatment. (C) Healed wound after 11 weeks.

Ischemic ulcers accounted for 20.57% of cases. The primary arterial intervention was angioplasty, with or without stenting. Reported procedures included superficial femoral artery angioplasty, tibial angioplasty, combined iliac stenting with superficial femoral artery angioplasty, tibial angioplasty with minor amputation, and angioplasty combined with radiofrequency ablation in selected cases. A representative ischemic ulcer following tibial angioplasty and minor amputation is shown in Figure 4.

**Figure 5:**
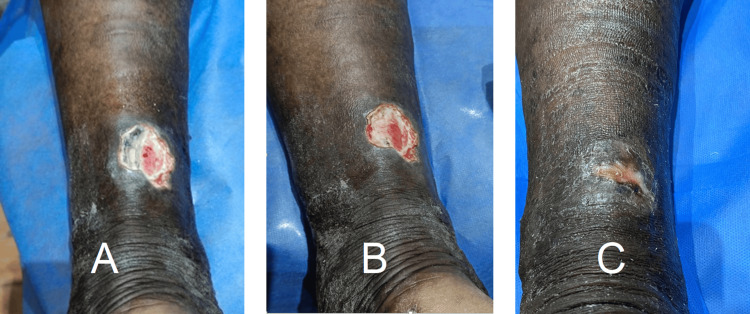
Healed traumatic ulcer after repeated trauma over the Achilles tendon The images show a traumatic ulcer healing in a male patient (56 years, group 3). (A) Traumatic leg ulcer over the tendon of Achilles’ at the start of treatment. (B) Wound healing after debridement and after three weeks of treatment. (C) Healed wound after 12 weeks (incomplete).

Traumatic ulcers were observed in three patients, of whom two were in the third treatment group, and all healed completely with a mean healing time of 7.6 weeks (Figure 5).

**Figure 6:**
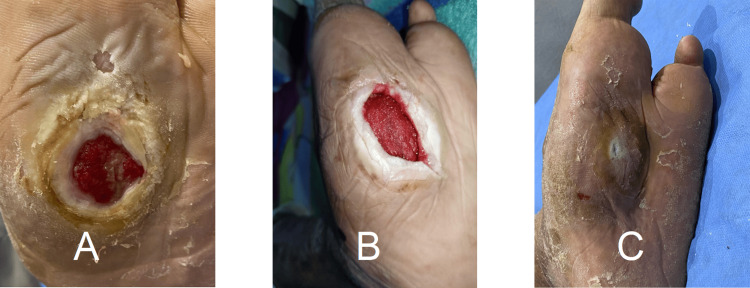
Healed pressure ulcer in Charcot foot The images show healing of the pressure ulcer in a male patient (66 years, group 3). (A) Pressure foot ulcer (Charcot’s foot) at the start of treatment. (B) Healing ulcer after debridement and after six weeks of treatment. (C) Healed ulcer after 12 weeks.

Pressure ulcers were present in seven patients. Complete healing occurred with mean healing times of 11.6 weeks in the first group, 14 weeks in the second group, and 9 weeks in the third group. A representative healed pressure ulcer in a patient with Charcot foot from the third group is shown in Figure 6.

Comparison of treatment arms demonstrated differences in healing duration and healing rates. The third group demonstrated the shortest mean healing time at 5.8 weeks, compared with 9.11 weeks in the second group and 9.73 weeks in the first group. Complete healing occurred in 10 (45.45%) patients in the first group, 25 (75.76%) patients in the second group, and 64 (approximately 94%) patients in the third group (Table 4).

**Table 4:** Healing rate, ulcer size, and mean healing time (mean ± SD) by treatment group

Group	Total patients	Total healing ulcers	Healing rate (%)	Ulcer size (mean ± SD, cm²)	Mean healing time (mean ± SD, weeks)
First group	22	10	45.45%	18.9 ± 0.6	9.73 + 0.092
Second group	33	25	75.76%	23.06 ± 0.4	9.11 + 1.02
Third group	68	64	94%	21.5 ± 0.7	5.8 + 0.12

Two-proportion Z-tests were performed to compare healing rates among treatment groups. Logistic regression analysis, using the first group as the reference category, demonstrated significantly higher odds of complete healing in both the second and third groups, with the strongest positive association observed in the third group (Tables 5, 6).

**Table 5:** Significance of healing rate comparisons Two-proportion Z-tests were performed to compare the healing rates between the groups: group 1 (antimicrobial topical therapy), group 2 (hyaluronic acid–based therapy), and group 3 (mixed combination therapy). The p-value was calculated to determine the significant difference between healing rates in different groups.

Groups	Z-value	P-value	Odds ratio (OR)	Significance
Group 1 versus group 2	2.2887	0.0221	3.7500	Significant (P < 0.05)
Group 1 versus group 3	3.0545	0.0023	4.6286	Highly significant (P < 0.01)
Group 2 versus group 3	0.4173	0.6765	1.2343	Not significant

**Table 6:** Logistic regression analysis of healing outcomes This table presents the results of a multivariable logistic regression model evaluating the association between treatment group and the likelihood of achieving complete ulcer healing within the study period. Group 1 (antimicrobial topical therapy) was used as the reference category. Coefficients represent the log odds of complete healing, with corresponding standard errors, z-statistics, p-values, and 95% confidence intervals. Groups 2 (hyaluronic acid–based therapy) and 3 (mixed combination therapy) demonstrated significantly higher odds of complete healing compared with group 1, with group 3 showing the strongest association.

Variable	Coefficient (β)	Std. error	z-statistic	P > |z|	95% CI
Constant (group 1)	-0.1823	0.428	-0.426	0.670	[-1.022, 0.657]
Group 2	1.3218	0.590	2.240	0.025	[0.165, 2.479]
Group 3	1.5322	0.523	2.931	0.003	[0.508, 2.557]

The proportion of patients reaching the secondary endpoint (incomplete healing within 16 weeks or withdrawal) was 17.89%. Secondary endpoint rates were highest in the first group and lowest in the third group (Table 7).

**Table 7:** Distribution of secondary endpoint (incomplete healing within 16 weeks or withdrawal) by treatment arm

Treatment arm	Secondary endpoint
First group	12 (54.55%)
Second group	7 (24.24%)
Third group	4 (6%)

**Figure 7:**
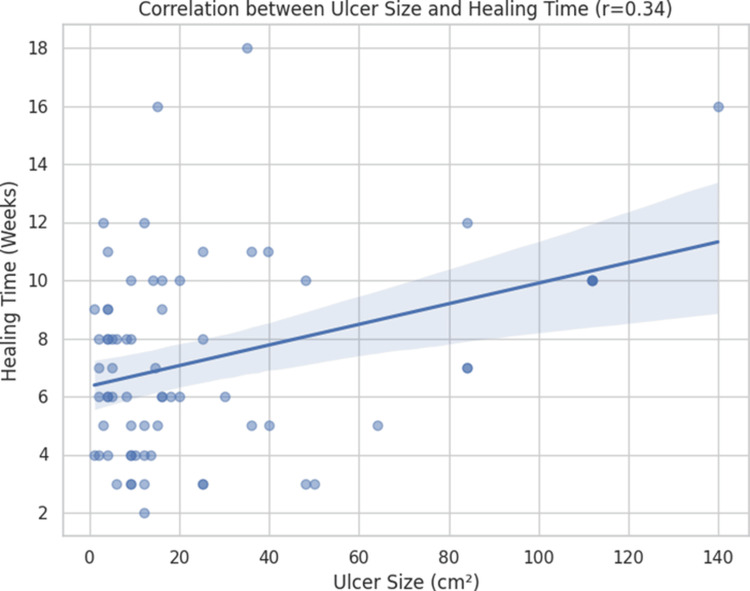
Correlation between initial ulcer size and healing duration The figure shows a positive correlation between ulcer size (cm2) and healing time (weeks). The (r) value of 0.34 indicates a weak to moderate positive linear relationship, and the p-value of 0.007 confirms that this relationship is statistically significant. The use of the symbol (r) in the title refers to the Pearson correlation test, which measures the linear association between two continuous variables.

Correlation analysis between initial ulcer size and healing duration demonstrated a correlation coefficient (r) of 0.34, indicating a weak positive correlation. This suggests that although larger ulcers tended to require longer healing times, treatment modality and patient-related factors played a more significant role. The correlation is illustrated in Figure 7.

The statistical analysis reveals a cohort with a high prevalence of diabetes mellitus with associated neuropathy, and a high incidence of ulcer infection. The most common etiology of ulceration is venous origin, followed by ischemic origin. The comparison of treatment arms suggests that arm 3 is associated with a significantly shorter mean healing time, despite having a comparable initial ulcer size to the other groups. The weak positive correlation between ulcer size and healing time suggests that treatment efficacy and patient comorbidities are likely more critical determinants of healing duration than initial ulcer size alone.

ANOVA was performed to compare continuous variables across the three treatment groups (Table 8). There was no statistically significant difference in baseline ulcer size between groups (p > 0.05), indicating comparable initial wound characteristics. In contrast, mean healing time differed significantly across treatment arms (p < 0.01), with the mixed combination topical therapy group demonstrating the shortest healing duration.

**Table 8:** Comparison of continuous variables across treatment groups (ANOVA) Values are presented as mean ± standard deviation. Comparisons between groups were performed using ANOVA. A p-value of <0.05 was considered statistically significant. ANOVA, one-way analysis of variance

Variable	Group 1 (n=22)	Group 2 (n=33)	Group 3 (n=68)	F-statistic values	P-value
Age (years)	47.5 ± 15	47.0 ± 12	51.6 ± 12.7	1.74	0.18
Ulcer duration before treatment (weeks)	22.2 ± 0.2	20.2 ± 0.3	18.9 ± 0.7	1.0502	0.3531
Initial ulcer size (cm²)	18.9 ± 0.6	23.06 ± 0.4	21.5 ± 0.7	0.1015	0.9036
Healing time (weeks)	9.73 ± 0.09	9.11 ± 1.02	5.8 ± 0.12	12.2438	<0.001

Subgroup analysis by ulcer etiology demonstrated heterogeneity in healing duration across treatment groups. Venous ulcers represented the majority of cases and showed significantly shorter healing duration in the mixed combination therapy group compared with groups 1 and 2 (p < 0.001). Arterial ulcers demonstrated a similar trend toward faster healing in group 3 (p = 0.0317). Mixed and pressure ulcers did not demonstrate statistically significant differences across treatment groups. Traumatic ulcers were rare (n = 3 overall), limiting meaningful statistical inference. These findings suggest that the superior healing outcomes observed in the combined formulation group were not confined to a single ulcer etiology but were most pronounced among venous ulcers. The analysis is demonstrated in Table 9.

**Table 9:** Subgroup aetiological ulcer analysis Subgroup p-value: calculated using Fisher’s exact test for the proportion of patients in each treatment group within the specific etiology compared to the rest of the study population. Total P-value: calculated using one-way analysis of variance for healing duration across the three treatment groups within each etiology. Odds ratio (OR): calculated for each subgroup relative to the other groups within the study population for that specific etiology. 95% CI: 95% confidence interval for the odds ratio using the Haldane–Anscombe correction.

Ulcer etiology	Patients n (%)	Healing duration	P-value	Odds ratio	95% CI
Traumatic ulcer in group 1	0 (0%)	NA	1.0000	1.18	0.06–24.40
Traumatic ulcer in group 2	1 (33.3%)	11.3 weeks	0.5518	1.99	0.25–15.88
Traumatic ulcer in group 3	2 (66.6%)	10.6 weeks	1.0000	0.84	0.11–6.64
Total traumatic ulcers	3 (100%)	10.8 ± 0.8 weeks	NS	NA	NA
Venous ulcer in group 1	3 (5.7%)	11.6 ± 0.6 weeks	0.1783	0.35	0.09–1.33
Venous ulcer in group 2	8 (15.2%)	11.2 ± 0.9 weeks	0.0344	0.36	0.14–0.93
Venous ulcer in group 3	43 (79.1%)	10.5 ± 0.07 weeks	0.0050	3.62	1.52–8.64
Total venous ulcers	54 (100%)	11.1 ± 0.5 weeks	<0.001	NA	NA
Arterial ulcer in group 1	3 (15%)	11.8 ± 1.2 weeks	0.4176	1.93	0.49–7.62
Arterial ulcer in group 2	6 (30%)	11.4 ± 0.9 weeks	0.5534	1.60	0.55–4.65
Arterial ulcer in group 3	11 (55%)	10.4 ± 0.8 weeks	0.2884	0.53	0.20–1.43
Total arterial ulcer	20 (100%)	11.06 ± 0.76 weeks	0.0317	NA	NA
Mixed etiology ulcer in group 1	2 (13%)	11.7 weeks	0.6456	1.67	0.36–7.65
Mixed etiology ulcer in group 2	4 (27%)	11.9 ± 0.7 weeks	0.7449	1.31	0.40–4.37
Mixed etiology ulcer in group 3	9 (60%)	9.9 ± 1.09 weeks	0.5634	0.70	0.23–2.09
Total mixed etiology ulcer	15 (100%)	10.9 ± 0.87 weeks	0.0087	NA	NA
Pressure ulcer in group 1	2 (30%)	11.6 weeks	0.1468	4.52	0.87–23.60
Pressure ulcer in group 2	4 (60%)	11.8 ± 0.6 weeks	0.0488	4.85	1.10–21.36
Pressure ulcer in group 3	1 (10%)	10.45 weeks	0.0052	0.10	0.02–0.60
Total pressure ulcer	7 (100%)	11.4 ± 0.6 weeks	0.2264	NA	NA

In the multivariable logistic regression model adjusting for treatment group, ulcer etiology was not independently associated with complete healing. Compared with venous ulcers (reference category), ischemic ulcers demonstrated lower odds of healing (adjusted OR 0.61, 95% CI 0.14-2.66, p = 0.514), although this did not reach statistical significance. Pressure ulcers showed similar healing odds to venous ulcers (adjusted OR 1.12, 95% CI 0.15-8.59, p = 0.91). Mixed etiology ulcers demonstrated a trend toward lower healing probability (adjusted OR 0.15, 95% CI 0.01-1.69, p = 0.125), though this was not statistically significant. The traumatic ulcer category produced an unstable estimate (adjusted OR > 99.9, p = 0.999), likely reflecting the very small number of traumatic cases in the cohort and model separation. Overall, ulcer etiology did not independently predict healing in the adjusted model, as shown in Table 10.

**Table 10:** Multivariable logistic regression analysis of ulcer etiology as an independent predictor of complete healing Adjusted ORs were derived from a multivariable logistic regression model with complete ulcer healing as the dependent variable. The model was adjusted for the treatment group. Venous ulcers were used as the reference category. A p-value of <0.05 was considered statistically significant. The extremely large OR observed for traumatic ulcers reflects sparse data and statistical instability due to the small number of cases. OR, odds ratio

Variable	Adjusted OR	95% CI	p-value
Ischemic vs venous	0.61	0.14–2.66	0.514
Pressure vs venous	1.12	0.15–8.59	0.91
Traumatic vs venous	>99.9	-	0.999
Mixed vs venous	0.15	0.01–1.69	0.125

## Discussion

CLUs remain a persistent therapeutic challenge because of their complex pathophysiology, frequent association with systemic comorbidities, and prolonged healing course. The present multicenter study provides a comparative evaluation of three topical wound care strategies and demonstrates that dressing selection significantly influences healing outcomes, time to ulcer closure, and overall treatment success.

The demographic characteristics of the study population are consistent with those reported in previous CLU cohorts, with a predominance of middle-aged patients and a high prevalence of diabetes mellitus and peripheral neuropathy, both of which are well-established risk factors for delayed wound healing and ulcer chronicity [5,6]. The prolonged mean ulcer duration prior to study inclusion reflects real-world referral patterns and highlights the refractory nature of ulcers managed in specialized wound care centers [7].

Venous ulcers constituted the majority of cases in this cohort, which aligns with epidemiological data identifying chronic venous insufficiency as the most common cause of lower-limb ulceration [4]. Importantly, etiological correction was achieved in a substantial proportion of patients through interventions such as compression therapy, venous ablation, or arterial revascularization. Current clinical guidelines emphasize that correction of the underlying pathology is fundamental to successful ulcer healing and should accompany any local wound treatment strategy [8,9]. The favorable overall healing rates observed in this study likely reflect adherence to these evidence-based principles.

The most significant finding of this study is the superior performance of the mixed combination treatment group receiving Zymalge gel. This group achieved the highest complete healing rate and the shortest mean healing time. Despite comparable baseline ulcer size across treatment arms, patients in the mixed combination group demonstrated faster wound closure, suggesting a beneficial effect on the wound microenvironment and tissue regeneration. Contemporary wound care concepts emphasize the importance of maintaining a balanced moist environment that supports cellular migration, angiogenesis, and extracellular matrix formation [10]. The observed outcomes are consistent with these biological principles.

In contrast, the antimicrobial gel group demonstrated the lowest healing rate and the longest duration to healing. Although antimicrobial dressings play an essential role in the management of clinically infected wounds, prolonged or indiscriminate use may negatively affect fibroblast activity and delay epithelialization [11,12]. These findings support current recommendations that advocate for targeted and time-limited antimicrobial use based on clinical signs of infection rather than routine long-term application [9,11].

The hyaluronic acid group demonstrated intermediate healing outcomes, reflecting the well-documented biological role of hyaluronic acid in hydration, modulation of inflammation, and promotion of granulation tissue formation [13]. However, the relatively higher rate of secondary endpoints in this group suggests that hyaluronic acid alone may be insufficient in complex or heavily colonized ulcers, particularly when additional antimicrobial or bioactive support is required.

The weak correlation observed between initial ulcer size and healing duration supports previous evidence indicating that ulcer size alone is a poor predictor of healing outcome [14]. Instead, healing appears to be influenced by a combination of factors, including etiological correction, infection control, optimization of the local wound environment, and systemic factors such as metabolic control and tissue perfusion.

From a clinical perspective, these findings emphasize that topical therapy selection should not be empirical but should instead be tailored to ulcer characteristics and integrated into a comprehensive, multidisciplinary management strategy. The superior outcomes associated with mixed combination therapy such as Zymalge gel suggest that it may represent an effective option for accelerating wound healing, potentially reducing treatment duration, healthcare resource utilization, and patient morbidity.

Several limitations should be acknowledged. First, the study followed a time-sequential allocation design rather than true randomization, which introduces the possibility of temporal bias. Seasonal variation in wound healing (e.g., differences in ambient temperature, humidity, or patient mobility between summer and winter months) may have influenced outcomes. Additionally, maturation effects cannot be excluded, as clinical proficiency and protocol familiarity may have improved over time across participating centers.

Second, unequal group sizes resulted from variability in patient recruitment across centers and time periods. Although baseline characteristics were comparable, this imbalance may introduce selection bias and influence statistical power. Also, the inclusion of venous, ischemic, pressure, traumatic, and mixed ulcers reflects the pragmatic real-world design of the study. However, heterogeneity of ulcer etiology may affect generalizability of dressing-specific conclusions. A particularly important limitation is the clustering of traumatic ulcers exclusively within the mixed combination therapy group. Traumatic ulcers generally have a more favorable healing trajectory compared with chronic venous or ischemic ulcers. All three traumatic ulcers healed within a mean of 7.6 weeks, which may have artificially inflated the healing rate and reduced the mean healing time in group 3. This represents a potential confounding factor that should be interpreted cautiously.

Third, the absence of blinding represents a methodological limitation. While complete epithelialization is an objective endpoint, the assessment of “absence of discharge” may carry some subjective interpretation, potentially introducing observer bias. Finally, although key baseline variables were comparable across groups, the quasi-experimental design limits the ability to fully exclude unmeasured confounding factors. These factors may warrant further investigation through larger, randomized, and blinded studies with extended follow-up periods.

## Conclusions

In conclusion, this study demonstrates that topical dressing choice influences healing outcomes in CLUs. Among the evaluated treatments, the mixed combination formulation (Zymalge gel), consisting of enzymatic antimicrobial components, alginate as a debridement agent, and hyaluronic acid for moisture regulation, was associated with superior healing outcomes. These findings support a more evidence-based and individualized approach to topical wound care within the broader framework of comprehensive ulcer management.
